# Can We Reduce Negative Blood Cultures With Clinical Scores and Blood Markers? Results From an Observational Cohort Study

**DOI:** 10.1097/MD.0000000000002264

**Published:** 2015-12-11

**Authors:** Svenja Laukemann, Nina Kasper, Prasad Kulkarni, Deborah Steiner, Anna Christina Rast, Alexander Kutz, Susan Felder, Sebastian Haubitz, Lukas Faessler, Andreas Huber, Christoph A. Fux, Beat Mueller, Philipp Schuetz

**Affiliations:** From the University Department of Internal Medicine, Medical Faculty of the University of Basel, Kantonsspital Aarau, Switzerland (SL, NK, DS, ACR, AK, SF, LF, BM, PS); Asclepius Medical Communications LLC, Ridgewood, NJ, USA (PK); University Clinic of Infectious Diseases, University Hospital Bern (SH); Institute of Psychology, University of Bern (LF); Department of Laboratory Medicine, Kantonsspital (AH); and Clinic of Infectious Diseases, Kantonsspital Aarau, Switzerland (CAF).

## Abstract

Only a small proportion of blood cultures routinely performed in emergency department (ED) patients is positive. Multiple clinical scores and biomarkers have previously been examined for their ability to predict bacteremia. Conclusive clinical validation of these scores and biomarkers is essential.

This observational cohort study included patients with suspected infection who had blood culture sampling at ED admission. We assessed 5 clinical scores and admission concentrations of procalcitonin (PCT), C-reactive protein (CRP), lymphocyte and white blood cell counts, the neutrophil-lymphocyte count ratio (NLCR), and the red blood cell distribution width (RDW). Two independent physicians assessed true blood culture positivity. We used logistic regression models with area under the curve (AUC) analysis.

Of 1083 patients, 104 (9.6%) had positive blood cultures. Of the clinical scores, the Shapiro score performed best (AUC 0.729). The best biomarkers were PCT (AUC 0.803) and NLCR (AUC 0.700). Combining the Shapiro score with PCT levels significantly increased the AUC to 0.827. Limiting blood cultures only to patients with either a Shapiro score of ≥4 or PCT > 0.1 μg/L would reduce negative sampling by 20.2% while still identifying 100% of positive cultures. Similarly, a Shapiro score ≥3 or PCT >0.25 μg/L would reduce cultures by 41.7% and still identify 96.1% of positive blood cultures.

Combination of the Shapiro score with admission levels of PCT can help reduce unnecessary blood cultures with minimal false negative rates.

The study was registered on January 9, 2013 at the ‘ClinicalTrials.gov’ registration web site (NCT01768494).

## INTRODUCTION

Although blood cultures are routinely collected in patients with suspected infection presenting to the emergency department (ED), their sensitivity for bacteremias is low, with <10% of cultures showing growth of bacteria.^1^ Moreover, contamination limits their specificity.^2^

Multiple studies have evaluated clinical scores for their utility in the prediction of bacteremia with the aim to improve the (pre-test) probability of positive culture results. A study conducted by Shapiro and colleagues enrolled 3730 ED patients with suspected infections and found 13 clinical parameters integrated into a single clinical score to be able to predict positive cultures with high accuracy.^3^ This score, which incorporated major and minor criteria, was also externally validated and proved to be a sensitive but not specific predictor of bacteremia.^4^ Another bacteremia prediction model proposed by Lee and colleagues found 7 clinical variables to accurately predict bacteremia in a total of 2422 patients with community-acquired pneumonia (CAP).^5^ Jones and colleagues studied 270 patients and found systemic inflammatory response syndrome (SIRS) criteria, the basis of the sepsis definition, to be predictive of bacteremia.^6^ Metersky and colleagues studied 13,043 patients with CAP and found the absence of recent antibiotic treatment, liver disease, 3 vital signs, and 3 laboratory abnormalities to be relatively accurate predictors of bacteremia.^7^ Finally, Tokuda and colleagues studied 526 patients with acute febrile illness and generated 3 different risk groups for bacteremia with 2 prediction algorithms (Tokuda scores I and II).^8^ The 5 clinical scores described above are summarized in full detail in Appendix 1.

In addition to the clinical scores discussed above, biomarkers that correlate with the probability of bacteremia have also been described. Several studies have found procalcitonin (PCT) levels to predict blood culture results in patients with pneumonia,^9–13^ urinary tract infections,^14^ sepsis,^15^ and acute febrile illness.^16^ Similar data are available for C-reactive protein (CRP),^13,17^ neutrophil-lymphocyte count ratio (NLCR),^18^ and lymphocytopenia,^18,19^ with significant differences in levels of these biomarkers between bacteremic patients and patients with negative blood cultures. Finally, red blood cell distribution width (RDW) has been proposed as a mortality marker for bacteremia.^20^

Most of these clinical scores have only been evaluated in patients with CAP,^5,7^ but not in a more heterogeneous, clinically challenging medical patient population presenting to the ED with suspected infection. We, therefore, aimed to validate the prognostic potential of these clinical scores alone and in combination with novel biomarkers in an ED patient population with suspected infection.

## METHODS

### Study Design and Setting

This is an observational cohort study. We prospectively included all consecutive medical patients with suspected infection presenting to the emergency department of a Swiss tertiary care hospital with additional regional primary and secondary care functions between February 2013 and October 2013 who had initial blood culture samples drawn. Blood cultures were drawn at the discretion of the treating physician. All patients were participants in the TRIAGE project, a prospective, observational study that aimed to devise an algorithm to optimize triage of adult patients with medical emergencies.^21,22^

The aim of this study was to compare 5 different clinical scores and 6 biomarkers for their ability to predict blood culture positivity. The primary endpoint was true blood culture positivity as assessed by 2 independent physicians and an infectious disease specialist according to Centers for Disease Control and Prevention (CDC) criteria (http://www.cdc.gov/getsmart/healthcare/implementation/clinicianguide.html).

Given that this was an observational quality control study, the Institutional Review Board (IRB) of the Canton of Aargau approved the study and waived the need for informed consent (approval number EK 2012/059). The study was registered at the “ClinicalTrials.gov” registration web site (NCT01768494).

### Participants and Definitions

Infections were classified on the basis of the main organ involved into the following categories: upper respiratory tract infections, lower respiratory tract infections, urinary tract infections, intra-abdominal infections, skin and soft tissue infections, central nervous system infections, endocarditis, foreign-material associated infections, and “other” infections. Excluded were patients who directly presented to the surgical ward and pediatric patients < 18 years of age.

In all patients, 2 pairs of blood culture samples for both aerobic and anaerobic cultures (equalling 40 mL of blood altogether) were collected before initiation of antibiotic therapy. Blood cultures were processed using an automated colorimetric detection system (BacT/ALERT, bioMérieux, Durham, NC).^23^ If blood culture bottles indicated bacterial growth, samples were Gram stained and subcultured. Identification of the pathogen was performed according to routine laboratory procedures. A blood culture was considered truly positive when it yielded a pathogen typical for the infection site. The evaluation was done by 2 independent physicians. In case of uncertainty, the case was discussed with an infectious disease specialist. The following species were usually considered to be contaminants: coagulase-negative Staphylococci, *Corynebacterium* species, and *Propionibacterium* species, unless an association with intravascular catheters/devices was suspected. In 1 case of a central line-associated infection and 1 case with clinical suspicion of endocarditis, infections with coagulase-negative Staphylococci were considered to be true infections (Appendix 2).

### Clinical Examination and Laboratory Data

In all patients, we recorded pertinent initial vital signs (eg, blood pressure and heart rate) and clinical parameters (eg, chills, vomiting, and comorbidities). Clinical information including socio-demographics, and patient outcomes were assessed prospectively until hospital discharge using the routinely gathered information from the hospital electronic medical system used for coding of Diagnosis-Related Group (DRG) codes.

Samples for later measurement of biomarkers were collected upon ED admission. The following markers were measured as part of routine care: CRP (normal range < 3.0 mg/L), albumin (normal range: 34–50 g/L), WBC (normal range: 4–10 × 10^9^/L), urea (normal range: 2.0–7.0 mmol/L, equals blood urea nitrogen [BUN] in mg/dL divided by 2.8), creatinine (normal range 80–115 μmol/L, divide by 88 for mg/dL), neutrophil percentage/proportion (normal range: 40–85%), neutrophil bands (normal range 0–10%), platelets (normal range 140–400 × 10^9^/L),^12,24^ plasma sodium (normal range: 136–146 mmol/L), and red blood cell distribution width (RDW) (normal range: < 15%). The neutrophil-lymphocyte count ratio (NLCR) was calculated by dividing the absolute neutrophil count by the absolute lymphocyte count.

In addition, we measured PCT levels post hoc with an automated rapid sensitive assay (KRYPTOR PCT; Thermo Scientific Biomarkers [formerly BRAHMS AG], Hennigsdorf, Germany; lower limit of detection: 0.02 μg/L).^25^

### Clinical Scores and Biomarkers

The clinical bacteremia scores including the Shapiro score,^3,4^ the Lee score,^5,6^ the SIRS criteria,^4,6^ the Metersky score,^9^ and the Tokuda score I and II^8,22^ are summarized in full detail in Appendix 1.

In addition, we also focused on several biomarkers that have been found to predict positive cultures. Biomarkers were used as continuous variables and at predefined cut-offs. First, we measured PCT using Kryptor technology and cut-off values were defined as 0.1 μg/L, 0.25 μg/L, 0.5 μg/L, and 1.0 μg/L based on previous studies.^9,12,23,24,26^ We used the published cut-offs of ≥ 10 and ≥ 12 for NLCR^18^ and < 1 × 10^9^ g/L for absolute lymphocytopenia.^27^ A RDW cut-off of >15 % was used as previously described.^20^

### Statistical Analysis

This report adheres to the STROBE guidelines for reporting observational studies.^28^ Discrete variables are expressed as counts (percentage) and continuous variables as medians and interquartile ranges (IQR). Frequency comparison was done by the chi square test. The 2-group comparison Mann–Whitney *U* test was used. To assess the prognostic performance of different parameters in predicting blood culture positivity, logistic regression analysis was used. We used biomarker levels as continuous variables and at predefined cut-offs as defined above. Logarithmic transformation of biomarker levels was used to obtain normal distribution for skewed variables. Receiver operating characteristics (ROC) were calculated, with the area under the curve being a measure of discrimination. The area under the ROC curve (AUC) is thus a summary measure over criteria and cut-point choices. The AUC summary equals the probability that the underlying classifier will score a randomly drawn positive sample higher than a randomly drawn negative sample. To test whether the biomarker levels improve clinical scores, we compared the nested logistic regression model with clinical scores and biomarkers with a model limited to the clinical scores alone. We also performed subgroup analyses to assess the performance of the different scores and markers within different types of infections and in Gram-positive and Gram-negative infections. We used STATA 12.1 (Stata Corp, College Station, TX). All testing was 2-tailed, with *P* <0.05 considered as indicating statistical significance.

## RESULTS

### Baseline Parameters

The median age of all included 1083 patients was 67 years (IQR 53–78) and 57.6% were males. True bacteremia was detected in 104 patients (9.6%). A detailed list of detected pathogens is presented in Appendix 2. A total of 28 patients (2.6% of all patients, 21.2% of those with positive blood cultures [28/132]) had contaminated blood cultures.

Table 1 shows patient characteristics on admission overall and separated according to blood culture results. Patients with positive cultures had less frequent antibiotic pretreatment and a lower diastolic blood pressure, whereas the core body temperature was significantly higher (38.5 °C vs 38.0 °C, *P* < 0.001). Laboratory analysis showed that CRP, NLCR, albumin, and urea were significantly higher in patients with positive blood cultures, whereas the lymphocyte count, RDW, and sodium were significantly lower. In addition, patients with positive blood cultures had almost 8.5-fold higher PCT levels compared to patients with negative cultures (1.69 vs 0.20 μg/L; *P* < 0.001).

**TABLE 1:**
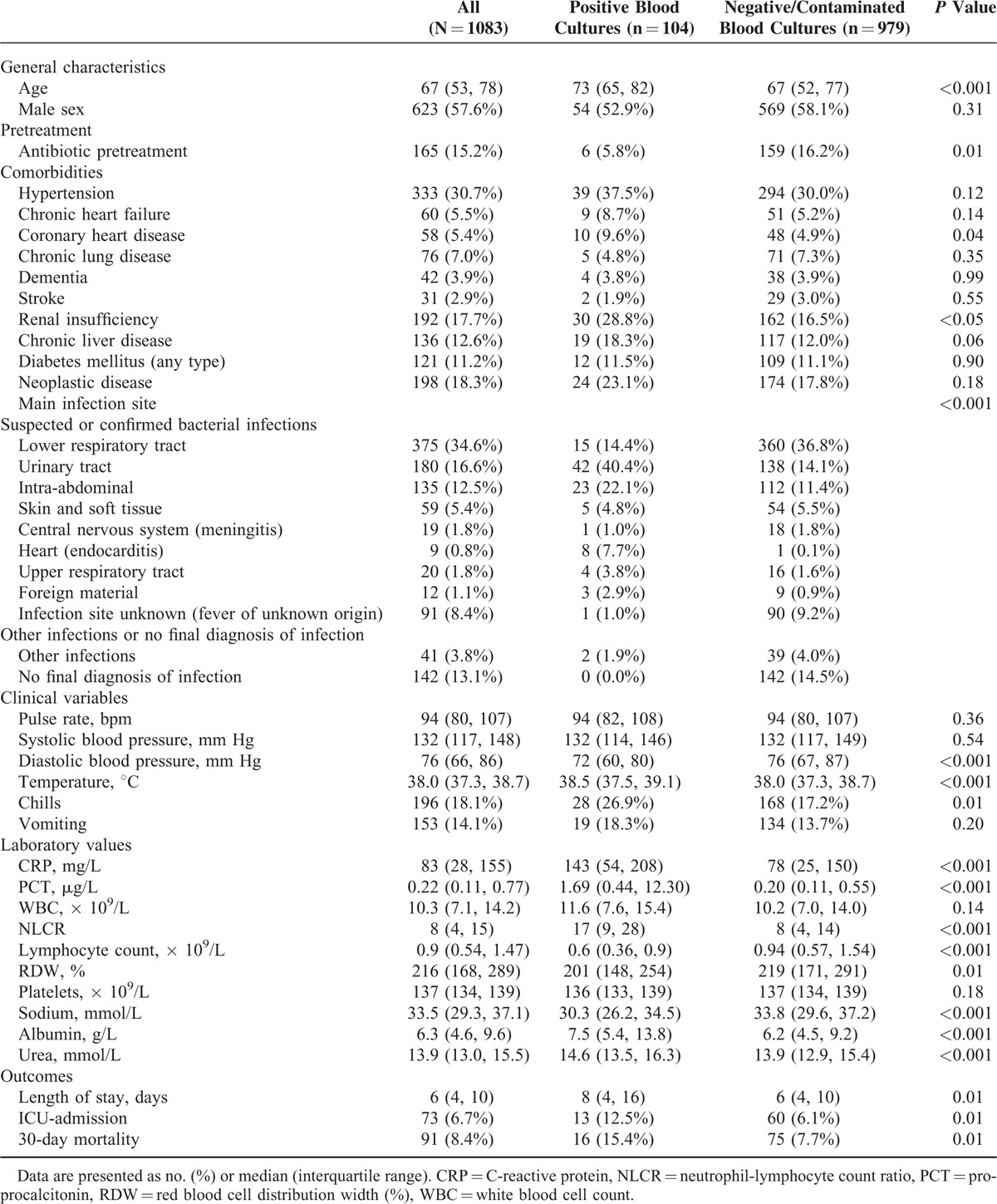
Baseline Characteristics of Patients Overall and Separated by Blood Culture Positivity and Negative or Contaminated Blood Cultures

As for in-hospital outcomes, patients with positive blood cultures were more frequently transferred to the ICU (12.5 % vs 6.1 %; *P* = 0.01), had an increased length of stay (8 days vs 6 days; *P* = 0.01), and had a significantly higher 30-day mortality rate (15.4% vs 7.7%; *P* = 0.01).

### Clinical Scores and Biomarkers to Predict Positive Blood Cultures

Table 2  displays the performance of the different clinical scores in predicting culture positivity from logistic regression models and discrimination (AUC). Of the clinical scores, the Shapiro score performed best with an AUC 0.729, followed by the Tokuda score II (AUC 0.665). The other clinical scores performed only moderately: Lee score (AUC 0.623), Metersky score (AUC 0.610), SIRS criteria (AUC 0.546), and Tokuda score I (AUC 0.566). Antibiotic pretreatment was a modest negative predictive factor for positivity of blood cultures, with an AUC of 0.552. The best biomarkers were PCT (AUC 0.803), NLCR (AUC 0.700), and lymphocyte counts (AUC 0.675). On the other hand, CRP, RDW, and WBC did not show significant results (AUC 0.645, 0.610, and 0.544, respectively).

**TABLE 2:**
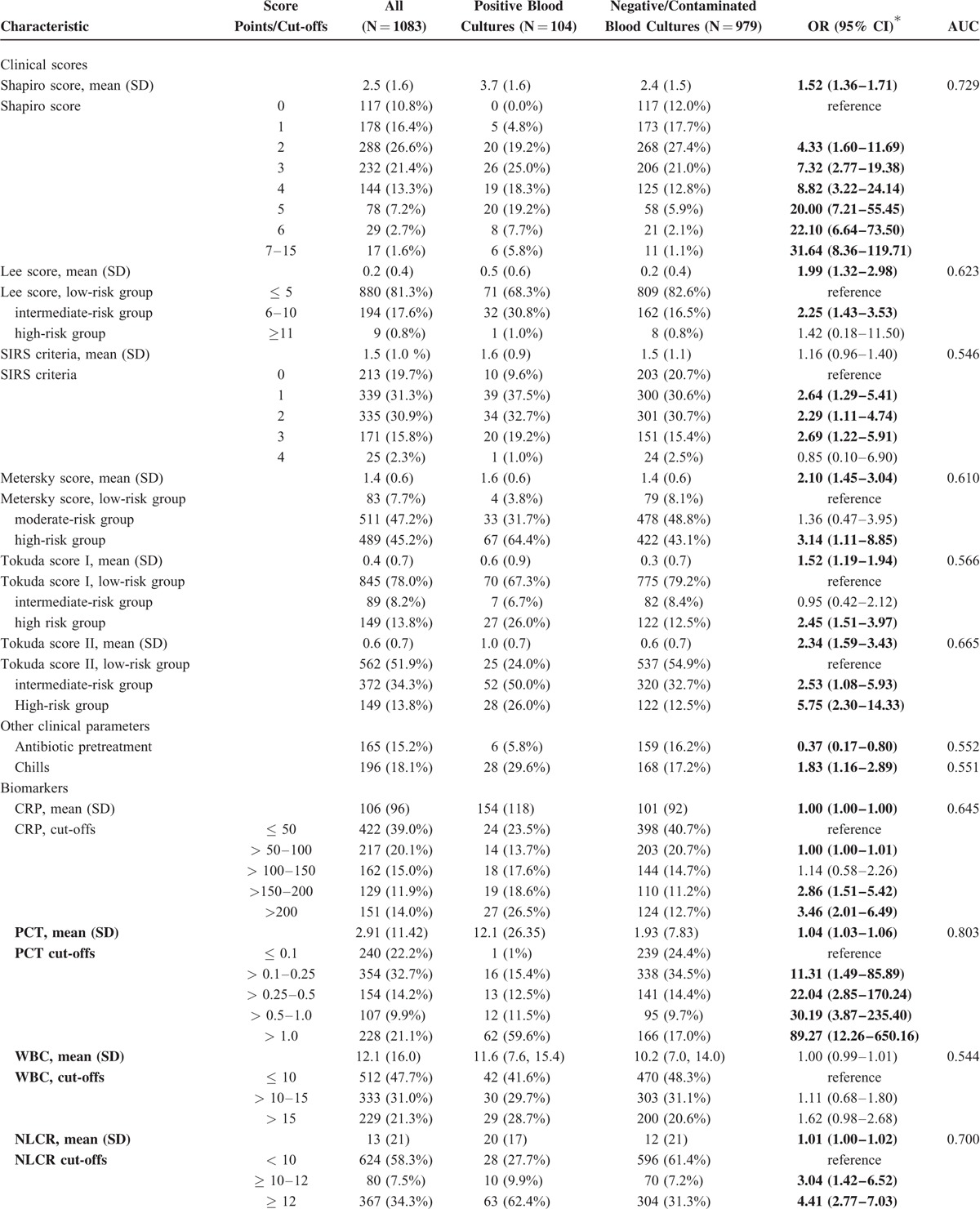
Results of Univariate Regression Analysis and AUC

**TABLE 2 (Continued):**
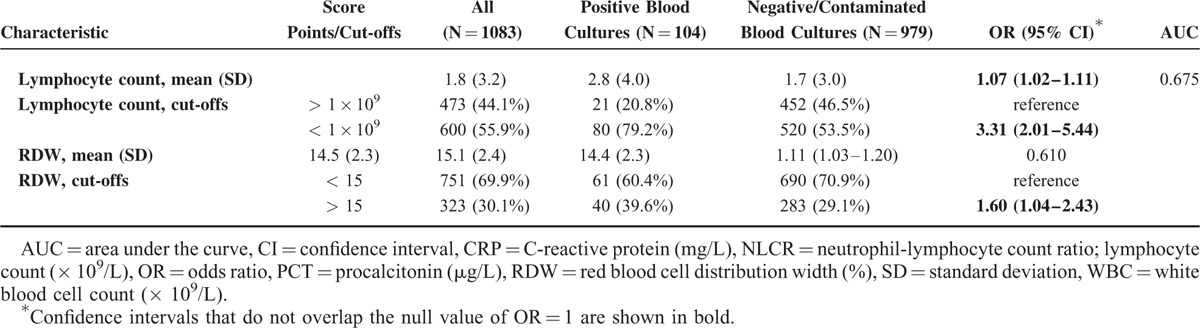
Results of Univariate Regression Analysis and AUC

### Combination of Clinical Scores and Biomarkers

Combining the Shapiro score and PCT showed the best results, with the AUC of the combined model being 0.827 (Table 3). The NLCR, CRP, or lymphocyte count could not significantly improve the predictive ability of the Shapiro score when combined individually with the latter. Combining the biomarkers PCT, NLCR, CRP, and lymphocyte count together with the Shapiro score resulted in an AUC of 0.817, which was not better than the combination of the Shapiro score and PCT alone.

**TABLE 3:**
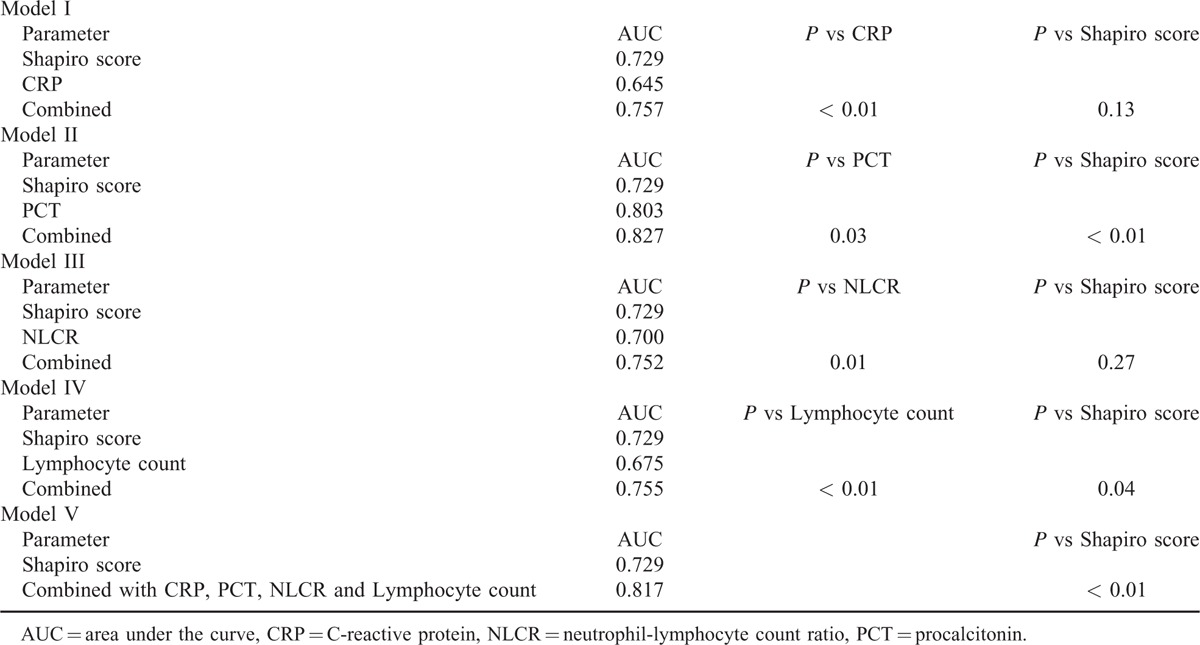
Combined Models

### Subgroup Analyses

Subgroup analyses (Table 4) showed the best performance of the Shapiro score to be for skin and soft tissue infections (AUC 0.756), urinary tract infections (AUC 0.694), and infections with Gram-negative bacteria (AUC 0.737). The Metersky score had the best performance for skin and soft tissue infections (AUC 0.737). On the other hand, PCT showed the highest AUC for lower respiratory tract infections and infections with Gram-negative bacteria, with AUC values of 0.876 and 0.837, respectively.

**TABLE 4:**
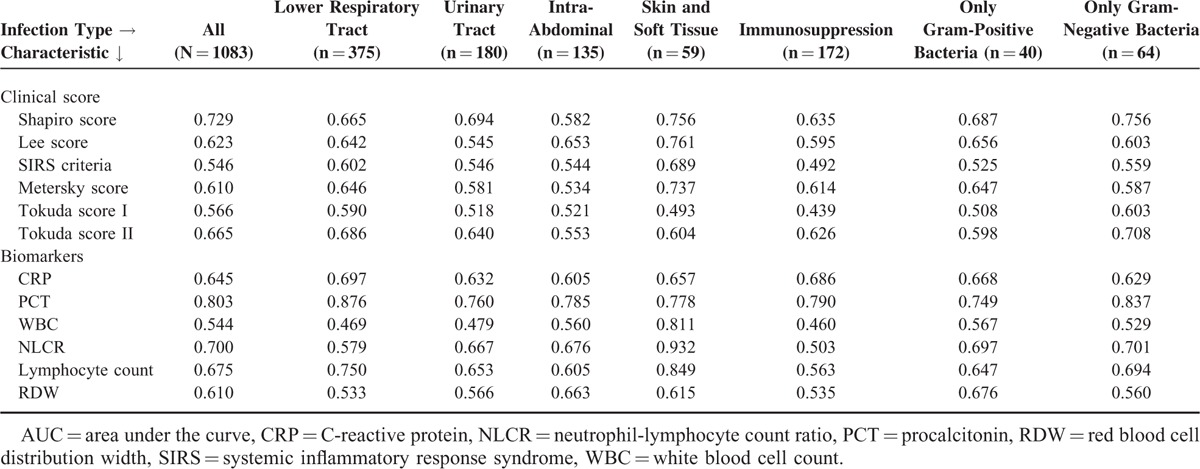
Subgroup Analyses (AUC)

### Diagnostic Measures

Table 5 shows the sensitivity, specificity, and positive and negative likelihood ratios for clinical scores, biomarkers, and promising combinations of the two. At a cut-off of ≥2 points as previously described by Shapiro and colleagues,^3^ the sensitivity of the Shapiro score for prediction of positive blood cultures was 95.2%. At a PCT cut-off of 0.1 μg/L, the sensitivity for prediction of positive blood cultures was 99.0%.

**TABLE 5:**
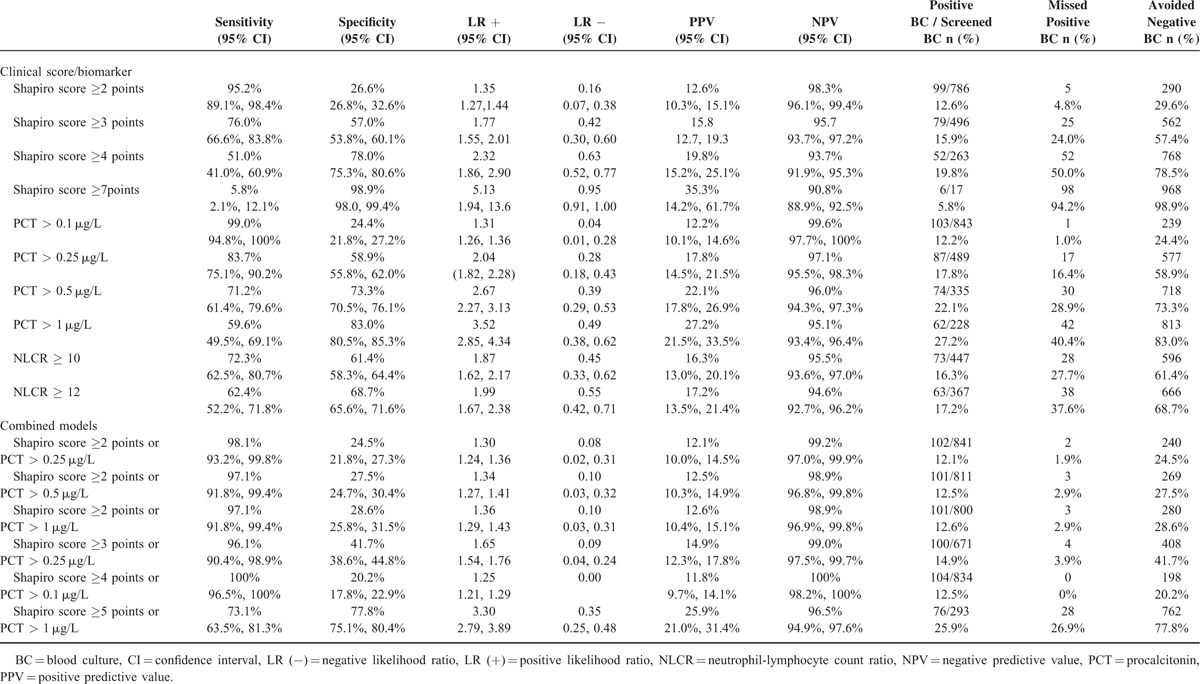
Diagnostic Performance for Prediction of Bacteremia by the Shapiro Score and Biomarkers

We also calculated diagnostic performance measures in a combined model using Shapiro's score and PCT. Limiting blood cultures to patients with either Shapiro scores ≥4 points or PCT levels > 0.1 μg/L would reduce negative sampling by 20.2% while still identifying 100% of positive cultures. Using Shapiro scores of ≥3 points or PCT levels > 0.25 μg/L would reduce negative sampling by 41.7% while still identifying 96.1% of positive cultures. (Appendix 3 provides additional details on the 4 patients [3.9%] missed with this algorithm). Finally, using a Shapiro score of ≥5 points or PCT > 1 μg/L would permit reduction of blood cultures by 77.8% and enable the identification of 73.1% of positive cultures.

## DISCUSSION

In this study that included consecutive medical patients presenting to the ED with suspected infection, we validated 5 clinical scores and biomarkers as potential predictors of bacteremia. The Shapiro score was the only score with good performance characteristics (AUC 0.729) which is in line with findings of a previous validation study.^4^ This score, when used in our patient population, would have helped reduce the number of blood cultures by 29.6% with minimal loss of sensitivity for true positive blood cultures, which is consistent with potential reduction of blood cultures by 27% in the original publication.^23^ Given that there were 1083 blood cultures drawn, a 29.6% reduction would result in ∼320 fewer cultures, with a potential cost saving of 46,400 US dollars [USD] (at an estimated cost of 145 USD for 2 sets of blood cultures per patient based on institutional data available at the University Hospital Basel). Additionally, this would reduce the number of false-positive cultures with subsequent investigations.

As for the biomarkers evaluated in this study, PCT proved to be the most reliable predictor of blood culture positivity, which is in line with previous research.^11^ Depending on the cut-off applied, PCT levels of < 0.1 μg/L identified patients with a low enough risk for bacteremia that unnecessary blood culture sampling could be avoided in these patients, resulting in significant financial benefits. With a PCT cut-off of > 0.1 μg/L, only 1 patient (1.0%) with positive blood cultures was missed whereas 24.4% of false negative blood cultures could have been avoided. Limiting blood cultures only to patients with increased PCT levels (ie, > 0.1 μg/L or > 0.25 μg/L) would likely result in significant cost benefits even when considering assay costs of ∼25 USD per PCT measurement.

Our study evaluating PCT in a broad and unselected ED patient population is novel since most other studies evaluated the accuracy of PCT to predict bacteremia in particular patient populations with specified focal infections such as pneumonia, urinary tract infections, or in patients with disseminated infections such as sepsis.^9–16^ To our knowledge, only 1 previous study has evaluated PCT in such a broad ED population with suspected infection.^29^ This study, which included an unselected patient population with suspected bloodstream infections, revealed that PCT levels were significantly elevated in patients with positive blood cultures and that PCT levels were significantly correlated with survival in patients with bacteremia. In our analysis, PCT was found to improve the predictive capabilities of the Shapiro score. To our knowledge, this is the first study to investigate the prognostic capacity of PCT in combination with the Shapiro score.

Our results are also in line with a previous ED-based study in Japan looking at the predictive value of different inflammation markers (PCT, CRP, WBC, platelets) to predict culture positivity.^30^ In this study using primary component analysis, PCT and platelets were found to be more helpful compared to CRP and WBC. Also, another recent study comparing the prognostic accuracy of PCT, CRP, and WBC in 513 patients presenting to the ED with signs/symptoms of local infections or sepsis found PCT to be the most accurate biomarker for diagnosis of sepsis and for mortality prediction.^31^

There exist no clear guidelines as to when blood cultures should be drawn.^1^ Because of the low yield of true positive results, routine sampling of blood cultures has been questioned.^1,3^ On the other hand, increasing antibiotic resistance calls for rapid bacterial identification and resistance testing to optimize treatment.^32^ There is thus an unmet need for accurate predictors of bacteremia. A higher pretest probability for true positive results would enable us to define a specific group of patients who would clearly benefit from blood cultures being drawn. Multiple clinical scores and biomarkers have been proposed to address this need. In this context, PCT and the NLCR have been shown to be the most promising ones.^18,33^

Although the vast majority of patients with positive blood cultures had PCT levels > 0.25 μg/L, 1 patient with a PCT level < 0.1 μg/L and 16 other patients with a PCT level < 0.25 μg/L also had positive, noncontaminated blood cultures. Appendix 3 provides additional details on the 17 patients with positive blood cultures but low PCT levels. Out of these, 5 patients [29.4%] suffered from endocarditis. Remarkably, 5 of 9 [55.6%] endocarditis cases with true positive blood cultures had relatively low PCT levels (0.15 μg/L to 0.24 μg/L). This finding that PCT is not routinely required to rule in or rule out infective endocarditis is in line with results of other studies.^34–36^ Another 8 of the 17 patients (47.1%) presented to the ED within < 24 h of symptom onset, and it is likely that PCT levels may not have reached their peak at this point in time. This hypothesis, however, needs to be evaluated in another prospective study.

In routine clinical practice, blood cultures are often drawn in response to fever.^1^ In our study population, however, 30 of the 104 patients with true positive blood cultures (28.8%) had temperatures < 38.0°C and 49 patients (47.1%) had temperatures < 38.5°C. This finding illustrates the limitations with regard to sensitivity and specificity of a single clinical parameter when used in isolation for decision making.

Our study has some limitations. First, the study was conducted at a single center, and the findings may not be readily applicable to other patient groups with different demographic characteristics. Second, due to the lack of clear guidelines concerning indications for obtaining blood cultures, some patients with fever and infections may not have had blood culture sampling on admission to the ED and are therefore not included in our study population. The decision to obtain the culture was left to the ED physicians’ clinical judgment or could even be made by the nursing staff with or without consultation with the clinician. Third, a potential limitation for widespread implementation of the Shapiro score combined with PCT levels > 0.1 μg/L is the large number of predictive factors included in the Shapiro score, which makes it complex and difficult to remember. However, the predictors themselves are routinely measured and available soon after gathering the medical history and physical examination in the ED setting. Determining PCT levels takes longer, however, with the need for point-of-care tests. Despite these limitations, the results of our study can be applied to a broad spectrum of internal medicine patients as the study was conducted in a relatively large number of patients.

Our study warrants future work in this area. A multicenter study to include a greater number of patients may help corroborate the findings of this study and reveal interesting new insights. Replication of this study in other facilities with patient populations having different demographic characteristics may also be informative. A study focusing on the application of our methodology to infections of specific body sites may be worthwhile, given that urinary tract infections and intra-abdominal infections were seen to have a higher risk for bacteremia (Table 1).

## CONCLUSIONS

In conclusion, although the Shapiro score is a useful clinical score on its own, combination of the Shapiro score with admission levels of PCT allows clinicians to abstain from ordering a significant number of potentially useless blood cultures, resulting in significant reductions in costs and false-positive results.

Based on the results of this study, a rational approach to blood culture collection may be as follows: for high-risk patients (eg, suspicion of endocarditis, immunosuppressed patients) blood cultures should be collected when ≥4 Shapiro criteria are fulfilled or when PCT levels are > 0.1 μg/L because the risk of false negative is minimal (0% in our study); for all other patients blood cultures should be collected when ≥3 Shapiro criteria are fulfilled or when PCT levels are > 0.25 μg/L as this cut-off reduces blood cultures by >40% with still a low risk of missing a positive culture (3.9%). In accordance with Shapiro and colleagues, we emphasize that careful clinical judgment must be used when applying a general clinical score to an individual patient and that the score should be overridden in instances in which specific circumstances and complex clinical conditions are present.^3^

## References

[1] Coburn B Morris AM Tomlinson G Does this adult patient with suspected bacteremia require blood cultures? *JAMA* 2012; 308:502–511. 22851117 10.1001/jama.2012.8262

[2] Schuetz P Mueller B Trampuz A Serum procalcitonin for discrimination of blood contamination from bloodstream infection due to coagulase-negative staphylococci. *Infection* 2007; 35:352–355. 17882355 10.1007/s15010-007-7065-0

[3] Shapiro NI Wolfe RE Wright SB Who needs a blood culture? A prospectively derived and validated prediction rule. *J Emerg Med* 2008; 35:255–264. 18486413 10.1016/j.jemermed.2008.04.001

[4] Jessen MK Mackenhauer J Hvass AM Prediction of bacteremia in the emergency department: an external validation of a clinical decision rule. *Eur J Emerg Med* 2014; [Epub ahead of print]. 10.1097/MEJ.0000000000000203 25222426

[5] Lee J Hwang SS Kim K Bacteremia prediction model using a common clinical test in patients with community-acquired pneumonia. *Am J Emerg Med* 2014; 32:700–704. 24856736 10.1016/j.ajem.2014.04.010

[6] Jones GR Lowes JA The systemic inflammatory response syndrome as a predictor of bacteraemia and outcome from sepsis. *QJM* 1996; 89:515–522. 8759492 10.1093/qjmed/89.7.515

[7] Metersky ML Ma A Bratzler DW Predicting bacteremia in patients with community-acquired pneumonia. *Am J Respir Crit Care Med* 2004; 169:342–347. 14630621 10.1164/rccm.200309-1248OC

[8] Tokuda Y Miyasato H Stein GH A simple prediction algorithm for bacteraemia in patients with acute febrile illness. *QJM* 2005; 98:813–820. 16174688 10.1093/qjmed/hci120

[9] Muller F Christ-Crain M Bregenzer T Procalcitonin levels predict bacteremia in patients with community-acquired pneumonia: a prospective cohort trial. *Chest* 2010; 138:121–129. 20299634 10.1378/chest.09-2920

[10] Schuetz P Amin DN Greenwald JL Role of procalcitonin in managing adult patients with respiratory tract infections. *Chest* 2012; 141:1063–1073. 22474148 10.1378/chest.11-2430

[11] Schuetz P Christ-Crain M Thomann R Effect of procalcitonin-based guidelines vs standard guidelines on antibiotic use in lower respiratory tract infections: the ProHOSP randomized controlled trial. *JAMA* 2009; 302:1059–1066. 19738090 10.1001/jama.2009.1297

[12] Schuetz P Christ-Crain M Muller B Procalcitonin and other biomarkers to improve assessment and antibiotic stewardship in infections—hope for hype? *Swiss Med Weekly* 2009; 139:318–326. 10.4414/smw.2009.12584 19529989

[13] Zhydkov A Christ-Crain M Thomann R Utility of procalcitonin, C-reactive protein and white blood cells alone and in combination for the prediction of clinical outcomes in community-acquired pneumonia. *Clin Chem Lab Med* 2015; 53:559–566. 25014522 10.1515/cclm-2014-0456

[14] van Nieuwkoop C Bonten TN van’t Wout JW Procalcitonin reflects bacteremia and bacterial load in urosepsis syndrome: a prospective observational study. *Crit Care* 2010; 14:R206. 21083886 10.1186/cc9328 PMC3220019

[15] Riedel S Melendez JH An AT Procalcitonin as a marker for the detection of bacteremia and sepsis in the emergency department. *Am J Clin Pathol* 2011; 135:182–189. 21228358 10.1309/AJCP1MFYINQLECV2

[16] Kim MH Lim G Kang SY Utility of procalcitonin as an early diagnostic marker of bacteremia in patients with acute fever. *Yonsei Med J* 2011; 52:276–281. 21319346 10.3349/ymj.2011.52.2.276 PMC3051230

[17] Schuetz P Suter-Widmer I Chaudri A Prognostic value of procalcitonin in community-acquired pneumonia. *Eur Respir J* 2011; 37:384–392. 20595156 10.1183/09031936.00035610

[18] Loonen AJ de Jager CP Tosserams J Biomarkers and molecular analysis to improve bloodstream infection diagnostics in an emergency care unit. *PLoS One* 2014; 9:e87315. 24475269 10.1371/journal.pone.0087315 PMC3903623

[19] Wyllie DH Bowler IC Peto TE Relation between lymphopenia and bacteraemia in UK adults with medical emergencies. *J Clin Pathol* 2004; 57:950–955. 15333656 10.1136/jcp.2004.017335 PMC1770434

[20] Yeh CF Chen KF Ye JJ Derivation of a clinical prediction rule for bloodstream infection mortality of patients visiting the emergency department based on predisposition, infection, response, and organ dysfunction concept. *J Microbiol Immunol Infect* 2014; 47:469–477. 23968756 10.1016/j.jmii.2013.06.012

[21] Schuetz P Hausfater P Amin D Biomarkers from distinct biological pathways improve early risk stratification in medical emergency patients: the multinational, prospective, observational TRIAGE study. *Crit Care (London, England)* 2015; 19:377. 10.1186/s13054-015-1098-z PMC4625457 26511878

[22] Schuetz P Hausfater P Amin D Optimizing triage and hospitalization in adult general medical emergency patients: the triage project. *BMC Emerg Med* 2013; 13:12. 23822525 10.1186/1471-227X-13-12 PMC3723418

[23] Thorpe TC Wilson ML Turner JE BacT/Alert: an automated colorimetric microbial detection system. *J Clin Microbiol* 1990; 28:1608–1612. 2116451 10.1128/jcm.28.7.1608-1612.1990 PMC267997

[24] Schuetz P Aujesky D Muller C Biomarker-guided personalised emergency medicine for all - hope for another hype? *Swiss Med Weekly* 2015; 145:w14079. 10.4414/smw.2015.14079 25695147

[25] Schuetz P Christ-Crain M Huber AR Long-term stability of procalcitonin in frozen samples and comparison of Kryptor and VIDAS automated immunoassays. *Clin Biochem* 2010; 43:341–344. 19747473 10.1016/j.clinbiochem.2009.08.029

[26] Schuetz P Chiappa V Briel M Procalcitonin algorithms for antibiotic therapy decisions: a systematic review of randomized controlled trials and recommendations for clinical algorithms. *Arch Intern Med* 2011; 171:1322–1331. 21824946 10.1001/archinternmed.2011.318

[27] de Jager CP van Wijk PT Mathoera RB Lymphocytopenia and neutrophil-lymphocyte count ratio predict bacteremia better than conventional infection markers in an emergency care unit. *Crit Care (London, England)* 2010; 14:R192. 10.1186/cc9309 PMC3219299 21034463

[28] von Elm E Altman DG Egger M The Strengthening the Reporting of Observational Studies in Epidemiology (STROBE) statement: guidelines for reporting observational studies. *Gac Sanit* 2008; 22:144–150. 18420014 10.1157/13119325

[29] Hattori T Nishiyama H Kato H Clinical value of procalcitonin for patients with suspected bloodstream infection. *Am J Clin Pathol* 2014; 141:43–51. 24343736 10.1309/AJCP4GV7ZFDTANGC

[30] Arai T Kumasaka K Nagata K Prediction of blood culture results by measuring procalcitonin levels and other inflammatory biomarkers. *Am J Emerg Med* 2014; 32:330–333. 24462198 10.1016/j.ajem.2013.12.035

[31] Magrini L Gagliano G Travaglino F Comparison between white blood cell count, procalcitonin and C reactive protein as diagnostic and prognostic biomarkers of infection or sepsis in patients presenting to emergency department. *Clin Chem Lab Med* 2014; 52:1465–1472. 24803611 10.1515/cclm-2014-0210

[32] Schuetz P Litke A Albrich WC Blood biomarkers for personalized treatment and patient management decisions in community-acquired pneumonia. *Curr Opin Infect Dis* 2013; 26:159–167. 23434895 10.1097/QCO.0b013e32835d0bec

[33] Howell MD Talmor D Schuetz P Proof of principle: the predisposition, infection, response, organ failure sepsis staging system. *Crit Care Med* 2011; 39:322–327. 21099424 10.1097/CCM.0b013e3182037a8e

[34] Singh M Koyfman A What is the role of procalcitonin in early diagnosis of infective endocarditis? *Ann Emerg Med* 2015; 66:25–26. 25109536 10.1016/j.annemergmed.2014.07.006

[35] Schuetz P Grolimund E Kutz A Procalcitonin-guided antibiotic therapy in patients with congestive heart failure and suspicion of lower respiratory tract infection: results from a randomized trial. *Crit Care* 2013; 17:S12.

[36] Schuetz P Kutz A Grolimund E Excluding infection through procalcitonin testing improves outcomes of congestive heart failure patients presenting with acute respiratory symptoms: results from the randomized ProHOSP trial. *Int J Cardiol* 2014; 175:464–472. 25005339 10.1016/j.ijcard.2014.06.022

