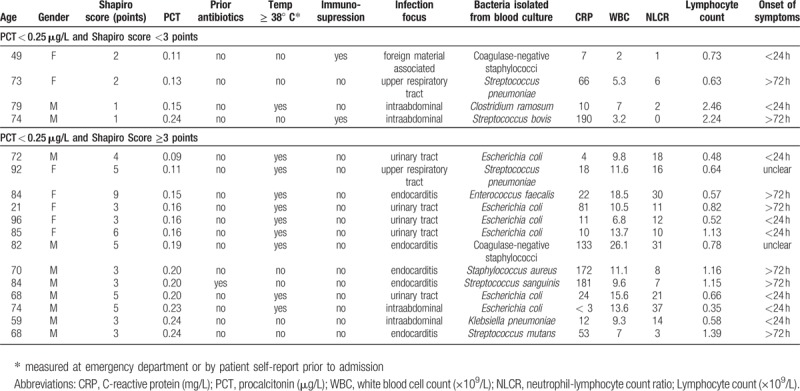# Can we reduce negative blood cultures with clinical scores and blood markers? Results from an observational cohort study: Erratum

**DOI:** 10.1097/MD.0000000000006197

**Published:** 2017-02-17

In the article “Can we reduce negative blood cultures with clinical scores and blood markers? Results from an observational cohort study”,^[1]^ which appeared in Volume 94, Issue 49 of *Medicine*, three appendices were referenced in the article, but no links to the appendices were provided. The appendices are provided below.

***Appendix 1:*** Clinical scores for prediction of positive blood cultures

*Shapiro score* [3,4]. This score includes “major criteria” (temperature ≥ 39.4°C, indwelling vascular catheter, or clinical suspicion of endocarditis) and “minor criteria” (temperature 38.3–39.3°C, age > 65 years, chills, vomiting, hypotension [systolic blood pressure < 90 mmHg], neutrophil proportion > 80%, WBC > 18 × 10^9^/L, neutrophil bands > 5%, platelets < 150 × 10^9^/L, and creatinine > 2.0 mg/dL or 176 μmol/L). According to the original study, a blood culture is indicated if at least one major criterion or two minor criteria are present. In addition to this cut-off, we categorized each patient on the basis of a sum score of 0–15 points derived by assigning one point per “minor criterion” and 2 points per “major criterion”.

*Lee score* [5]. Lee and colleagues found systolic blood pressure < 90 mm Hg, heart rate > 125 /minute, body temperature < 35°C or > 40°C, WBC < 4 × 10^9^/L or > 12 × 10^9^/L, platelets < 130 × 10^9^/L, albumin < 3.3 g/dL or 33 g/L, and CRP > 17 mg/dL or 170 mg/L to accurately predict bacteremia in patients with CAP. As proposed by the authors, we assigned five points for body temperature <35°C or >40°C, three points each for hypotension, tachycardia, or CRP > 17 mg/dL, and 2 points each for WBC < 4 × 10^9^/L or > 12 × 10^9^/L, or albumin < 3.3 g/dL. The overall risk score was regarded as the sum of the calculated points, with the following risk categories being recognized: low risk: ≤ 5 points, moderate risk: ≥6–10 points, and high risk: ≥11 points.

*SIRS criteria* [6]. As suggested by Jones and Lowes, who proposed that systemic inflammatory response syndrome (SIRS) could serve as a predictor of bacteremia, one point was assigned to each SIRS criterion: temperature >38.3°C or <36°C, heart rate ≥ 90 /min, tachypnea with a respiratory rate ≥ 20 breaths/min, WBC > 12 × 10^9^/L, or normal WBC with ≥10% immature (band) forms.

*Metersky score* [7]. This score includes antibiotic treatment, liver disease, systolic blood pressure < 90 mm Hg, temperature < 35°C or ≥40°C, pulse ≥ 125 /min, BUN ≥ 30 mg/dL or urea ≥ 10.7 mmol/L, sodium < 130 mmol/L, and WBC < 5 × 10^9^/L or >20 × 10^9^/L. As recommended in the original publication, the risk of bacteremia was evaluated according to the number of predictors present: low-risk-group with zero predictors and prior antibiotics, moderate-risk group with zero predictors and no prior antibiotics or with one predictor and prior antibiotics, and high-risk group with one predictor and no prior antibiotics or with two or more predictors.

*Tokuda score I and II* [8]. For Tokuda score I, the presence of chills, pulse > 120 /min, and high risk infective site was evaluated and patients were classified as follows with regard to their risk for bacteremia: low risk (no chills and pulse < 120 /min or chills and physicians’ diagnosis of low risk infective site), intermediate risk (no chills and pulse > 120 /min), and high risk (chills and physicians’ diagnosis of high risk infective site) as proposed in the original article. The Tokuda score II was calculated similarly, except that the variable pulse was replaced by CRP and the following risk groups were recognized: low risk (no chills and CRP < 10 mg/dL or 100 mg/L or chills and physicians’ diagnosis of low risk infective site), intermediate risk (no chills and CRP > 10 mg/dL or 100 mg/L), and high risk (chills and physicians’ diagnosis of high risk infective site).

***Appendix 1:*** Clinical scores for prediction of positive blood cultures

**Figure:**
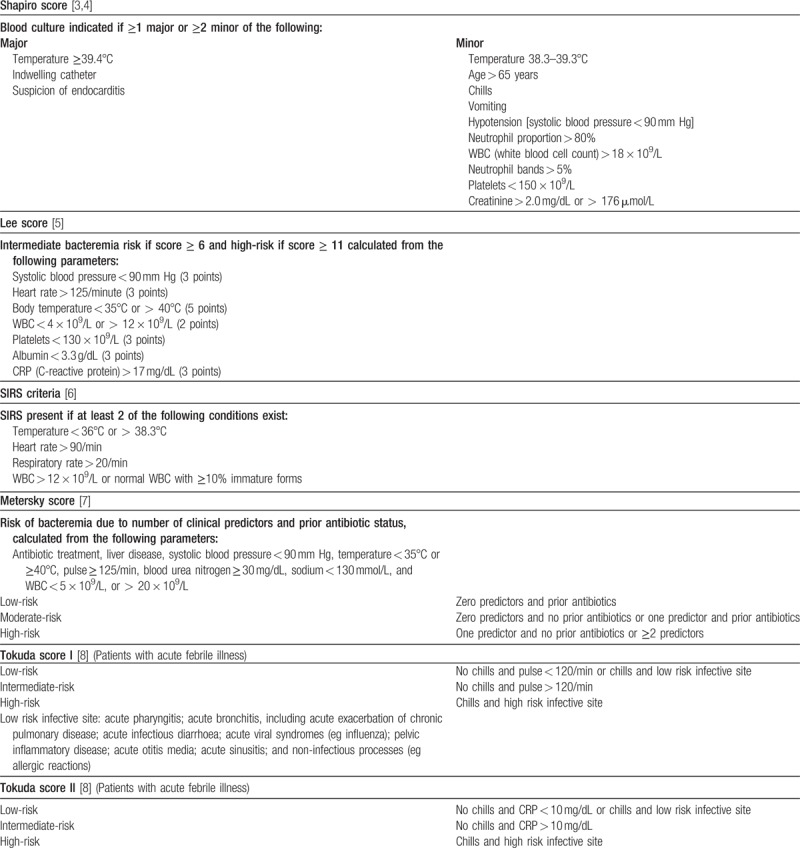


***Appendix 2**:* Microorganisms isolated from blood cultures

**Figure:**
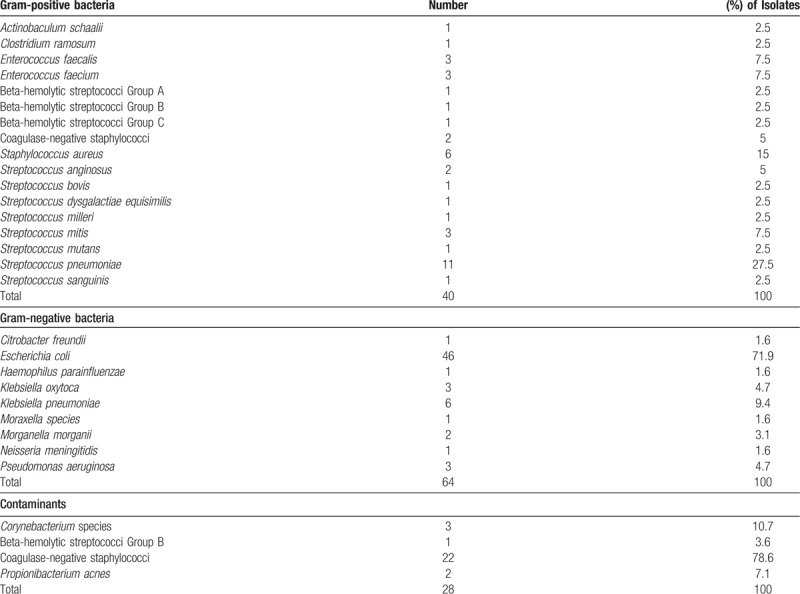


***Appendix 3:*** Characteristics of patients with true positive blood cultures and PCT < 0.25 μg/L

**Figure:**